# Guardians of Memory, Dignity, and Family Cohesion: The Enduring Protective Role Underpinning Suicide-Bereaved Mothers’ Psychosocial Needs and Engagement with Mental Health Care

**DOI:** 10.3390/nursrep16080262

**Published:** 2026-07-29

**Authors:** Rafailia Zavrou, Andreas Charalambous, Evridiki Papastavrou, Anna Koutroubas, Maria Karanikola

**Affiliations:** 1 Cyprus Mental Health Services, Department of Nursing, Cyprus University of Technology, Limassol 3041, Cyprus; rc.zavros@edu.cut.ac.cy; 2 Department of Nursing, Cyprus University of Technology, Limassol 3041, Cyprus; andreas.charalambous@cut.ac.cy (A.C.); e.papastavrou@cut.ac.cy (E.P.); akoutrouba@hotmail.com (A.K.)

**Keywords:** suicide bereavement, maternal grief, needs, child loss, Cyprus

## Abstract

**Background/Objectives**: While there is an existing body of quantitative data on the psychological and mental burden of suicide-bereaved parents, further qualitative research is needed to explore suicide-bereaved mothers’ living experiences in specific sociocultural contexts, especially in Southern Europe and the Eastern Mediterranean. Given the persistent stigma surrounding suicide in these societies, and the fact that previous research has often overlooked mothers’ perspectives in favor of broader samples of bereaved parents, we explored the psychosocial needs of Greek-speaking suicide-bereaved mothers in the Republic of Cyprus, and their experiences in accessing formal mental healthcare support. **Methods**: An inductive, secondary content analysis of qualitative data collected through personal semi-structured interviews with ten suicide-bereaved mothers was employed. **Results**: Participants’ psychosocial needs centered around a “persistent orientation towards protection,” encompassing three interconnected domains: (1) self-protection and the need for acceptance by shielding themselves from stigma, social judgment, and emotional disintegration, (2) ensuring a safe and protective environment for the surviving family by safeguarding the psychological well-being and cohesion of surviving family members, and (3) protecting the posthumous dignity and memory of the deceased child. Rather than seeking formal support, participants overwhelmingly avoided mental health services, citing a lack of empathy, cultural misunderstanding, and fear of further stigmatization. Mental health professionals were often perceived as inadequate or even harmful, undermining participants’ need for protective attitudes, self-reliance and self-respect during bereavement. These responses reflected how stigma and gendered social expectations surrounding suicide shaped participating bereaved mothers’ disengagement from the healthcare system, despite their intense psychological needs. **Conclusions**: These findings underscored how gendered social expectations, combined with the stigma surrounding suicide, created significant psychosocial barriers to mental health care for women navigating traumatic grief, particularly in sociocultural contexts where suicide remains highly stigmatized.

## 1. Introduction

Suicide is the fourth leading cause of death among people aged 15–29 [1]. The death of a child or young person by suicide is one of the most traumatic and devastating experiences for the surviving family and friends [2,3]. Evidence suggests that suicide-exposed family members are more likely to have psychological, mental and physical health problems, including an increased risk of hospitalization for psychiatric care, relative to the general population. Related vulnerabilities [4,5,6], including depression, self-harm, suicidal thoughts, and death by suicide, are more prevalent among partners and parents of the deceased [4,7].

Furthermore, those who have lost a loved one to suicide are 80% more likely to leave their job or their academic studies than those who have lost a loved one to sudden death from natural causes [6]. This illustrates the impact of suicide on the bereaved survivors at both educational and occupational levels, severely affecting their functioning. Other data underline shame, rage, and existential challenges experienced by suicide-bereaved parents [8]. Additionally, quantitative research comparing scores on grieving dimensions has shown that suicide-bereaved parents report higher stigma scores than other bereaved parent groups [9].

Despite the enduring suffering, suicide-bereaved parents are more likely to receive less help from family and friends and experience delays in receiving formal support than those who have lost a loved one to any other types of unexpected death [9,10,11]. Qualitative studies from the United Kingdom [12], Australia [13], and African-American communities [14] highlight that stigma is a major obstacle in seeking formal help and obtaining appropriate support during suicide bereavement. Pitman et al. [6] critically demonstrated cultural differences in how stigma is perceived following sudden deaths, with stigma and self-blame being linked to complicated grieving processes and ineffective recovery of the bereaved. Similarly, the support needs of suicide-bereaved parents may not be generic either, even within the same cultural community [6]. Thus, a deeper comprehension of how the death of a child or a young person by suicide has inflicted mothers’ unmet psychosocial needs and attitudes towards seeking support may inform interventions aimed at promoting effective gender-specific help-seeking, evidence-based person-centered care, and updating relevant mental health services.

At the same time, research has often overlooked mothers’ perspectives, grouping them together with fathers or with more general samples of “bereaved parents” [15,16,17]. Although qualitative studies have explored parental suicide bereavement, mothers’ experiences have most often been examined alongside those of fathers, limiting understanding of their potentially distinct psychosocial and support needs and interactions with formal mental healthcare services [15,16,17]. Yet, mothers are frequently positioned as primary caregivers and held responsible for managing the needs of the rest of the family, as well as maintaining family cohesion [18]. Subsequently, they may face heightened stigma, self-blame, and social judgment when a child dies by suicide, as well as unique pressures to support surviving family members [2]. Thus, their experiences, psychosocial needs, and barriers to support deserve focused exploration.

The Republic of Cyprus (RC) is a small Eastern Mediterranean country, characterized by a sociocultural setting shaped by deep family ties, an honor culture and religious traditions [19]. According to Christian Orthodox Christianity, which is the prevailing religion in the RC [19], suicide is frequently viewed as a moral and religious offence [20,21]. Thus, the stigma around suicide remains severe, frequently leading to silence and secrecy within families and communities [21]. Subsequently, suicide-bereaved individuals are most of the time reluctant to seek support to avoid further stigmatization [22], while access to specialized grieving services is also limited in the RC. As a result, the informal family support network is the most common form of grieving support “service” in the RC [21].

These cultural and systemic issues strongly influence how suicide-bereaved mothers grieve and seek support [23], emphasizing the significance of assessing their support expectations in this particular socio-cultural context, as well as the dynamic between them and formal support services [24]. Nevertheless, most of the existing data are quantitative, providing a broad understanding of the impact of suicide on bereaved survivors, mainly in relation to grief severity, post-traumatic, depressive and anxiety symptoms; yet key questions about mothers’ specific priorities and psychosocial needs remain unexplored [25,26,27,28,29]. Subsequently, further research is still needed to exclusively explore bereaved mothers’ self-perceived psychosocial needs and challenges in receiving mental healthcare within diverse sociocultural contexts, especially in Southern Europe and the Eastern Mediterranean region, where both gender- and stigma- related issues are culturally specific [30].

In particular, there are only limited studies on the self-perceived psychosocial needs of suicide-bereaved mothers outside of central European cultures [31,32]. Subsequently, the dynamics surrounding the consequences of a child’s suicide on bereaved mothers remain unclear in these contexts [8]. Nevertheless, mothers’ experiences of suicide bereavement are shaped not only by the loss itself but also by the intersection of gendered caregiving expectations, suicide-related stigma, and the responsiveness of healthcare systems [16]. Overall, a deeper understanding of the interplay between suicide-bereaved mothers’ self-perceived psychosocial needs and their social context may advance supportive psycho-therapeutic interventions in mental healthcare services towards this vulnerable group.

Within this framework, the present study undertook a secondary qualitative analysis of data on the living experience of Greek-speaking suicide bereaved mothers in the RC. In particular, this study builds on an existing qualitative dataset [2] to address a previously unexplored topic; thus, the aim of this study was to explore a distinct research question concerning suicide bereaved mothers’ self-perceived psychosocial needs and experiences of accessing formal mental healthcare. By re-examining the original narratives through a new analytic lens, this study seeks to deepen understanding of how bereaved mothers navigate suicide loss outside formal therapeutic settings, including their psychosocial concerns and priorities, as well as their experiences of interacting with institutional settings.

Given the persistent stigma surrounding suicide in Eastern Mediterranean and Southern European societies, such as the RC, the present findings may contribute to the development of culturally responsive, stigma-sensitive and gender-informed postvention services tailored to mothers following the suicide of a child. Nevertheless, mothers bereaved by suicide may face unique psychosocial challenges to accessing mental health care [2,16,23]. These challenges may not only be shaped by cultural silence and societal judgment related to suicide, but also by a healthcare system that may inadequately respond to their gendered and grief-specific concerns [33]. Indeed, previous research suggests that fathers and mothers may grieve in different ways, which indicates the need for differentiated therapeutic approaches [34]. This underscores the importance of generating data that can inform the development of targeted interventions specifically addressing the emotional support priorities and needs of bereaved mothers. In light of these challenges, this study is expected to contribute to understanding how suicide-bereaved mothers navigate loss outside of formal therapeutic settings, shedding light on their psychosocial needs and the institutional challenges they encounter. Thus, this new insight is expected to inform how suicide-bereaved mothers may be effectively supported by formal mental healthcare services across the lifespan based on their personal experiences following the loss of their child.

## 2. Materials and Methods

### 2.1. Study Design

An inductive, secondary content analysis of data collected primarily by Zavrou et al. [2] was employed. An interpretivist epistemological perspective, which assumes that meanings are socially constructed and subjective, informed the present study. This paradigm allows researchers to understand and reveal the context-specific processes by which individuals make sense and provide insight into their lived experience through their narratives [35]. Accordingly, this secondary analysis did not seek to verify objective truths or to verify predefined assumptions. Instead, the focus was on generating a deeper understanding of how suicide-bereaved mothers interpreted their psychosocial needs and experiences with formal healthcare services. Consistent with this perspective, this inductive qualitative content analysis allowed categories and sub-categories to emerge from participants’ narratives rather than being imposed through predefined theoretical frameworks [36,37,38,39].

Consequently, a new research question was articulated for the purpose of the present study, and a new analysis was conducted on the raw data to go beyond the original study by Zavrou et al. [2], constituting a conceptually distinct investigation. Specifically, the focus of the previously published study by Zavrou et al. [2] was on the exploration of the living experience of suicide bereaved parents, with a focus on their interpretations concerning their child’s suicide, its aftereffects and their coping strategies. In contrast, the present new research question emerged from gaps in understanding the specific self-perceived psychosocial needs of suicide-bereaved mothers and the dynamics of receiving formal mental healthcare support. These topics were not systematically analyzed or reported in the publication by Zavrou et al. [2]. Thus, a relevant, new research question was framed and a secondary to the primarily data analysis was employed [36].

Importantly, although the interviews were originally conducted to explore the broader living experience of suicide-bereaved parents, the interview guide included extensive discussion of participant mothers’ emotional responses, coping, support experiences and perceived needs. During the primary analysis, the richness of participant mothers’ narratives regarding unmet psychosocial needs became evident but remained beyond the scope of the original publication. Therefore, a secondary analysis was undertaken to address this distinct research question using the original verbatim transcripts. Because the data contained substantial unexplored material directly relevant to the new research aim, secondary analysis was considered both methodologically appropriate and ethically justified. Rather than reusing findings, the present secondary analysis involved a completely new coding process focused specifically on mothers’ self-perceived psycho-social needs and experiences of accessing formal care. Hence, the original verbatim transcripts were re-examined through a new inductive coding process guided by this distinct research question, resulting in new categories and interpretations beyond those presented previously.

Overall, the present secondary analysis was deemed the most appropriate methodological approach, as it enabled further efficient use of already available resources while generating new substantive insights [36,37,38]. This approach also reduced the burden on both participants and researchers, a benefit particularly important in studies of sensitive topics and/or hard-to-reach communities, such as suicide-bereaved mothers, where primary data collection poses significant challenges [38,39]. The present secondary analysis was conducted openly to let new insights emerge from the data itself as it is defined by the inductive method [40,41].

Reporting of the current findings followed the Consolidated Criteria for Reporting Qualitative Research (COREQ) checklist (please see Supplementary Table S1) [42].

### 2.2. Aim and Objectives

The aim of this study was to assess the experiences of Greek-speaking mothers in the RC whose child died by suicide, with a focus on their interpretations about (a) their psycho-social needs, and (b) accessing formal mental health care.

### 2.3. Participants and Recruitment

There is no formal suicide registry in the RC to provide identifying information on those who have died by suicide or of their surviving relatives. As a result, the researchers could not directly access the target population. Nor are there support or self-help groups for parents who have lost a child to suicide in public or private mental health services or provided by non-governmental organizations in the RC.

To address these challenges, a formal communication strategy was developed to inform both public and private mental health services about the study’s objectives and methods. This approach aimed to reach the entire target population and recruit as many participants as possible. As a result, participants were partially recruited through the mental health services used by suicide-bereaved mothers or the deceased. Due to the absence of a formal registry of families affected by suicide, it was not possible to estimate the size of this community accurately.

To attract volunteers from diverse backgrounds, the study was also advertised through media outlets, at pertinent scientific meetings and on social media platforms, e.g., Facebook and Twitter, with postings directed at groups and pages concerned with mental health services and medical topics. Participants were encouraged to share information about the study within their own networks. This open call, along with a snowball sampling technique, effectively broadened the purposive sampling pool of eligible individuals.

Participants either contacted the research team directly to express their interest (via phone) or allowed the researcher to contact them using the information they provided, depending on how they learned about the study.

### 2.4. Data Collection

Data were collected (July 2017–August 2019) by RZ and MK. The extended data collection period reflected the sensitive nature of the study and the challenges associated with recruiting this hard-to-reach population, since there is no national registry or centralized database of suicide-bereaved family members in the RC to facilitate participant identification. Consequently, recruitment relied on purposive sampling through multiple referral pathways and required sufficient time to identify eligible participants and arrange interviews according to their emotional readiness and availability.

#### 2.4.1. Sampling Method

Once participants provided written informed consent to participate in the study, the primary author assessed their alignment with the following inclusion criteria: (a) being a mother of a son or a daughter who died by suicide; (b) fully understanding the study’s purpose and procedures; (c) having the ability to reflect on the personal experience of the loss, and (d) to communicate these experiences to the researchers. There was no specific inclusion criterion regarding the time passed since the son’s/daughter’s suicide. There were no exclusion criteria. Participants were recruited through various ways: six individuals were referred through a member of their treatment team, two were recruited through social media, and the remaining two found out about the study through their peers.

#### 2.4.2. Sample Size

For the present secondary analysis, no additional participant recruitment was undertaken. Instead, the desired sample was achieved through repeated, iterative examination of the existing dataset until no further concepts or interpretations relevant to the new research question emerged. Specifically, during the concurrent analysis and data collection, the sample size was determined to be 10 participants, based on theoretical and data saturation criteria. Specifically, data analysis suggested that the recruitment of new participants was complete when no new themes emerged. At that point, all recruitment of participants stopped at that point. Due to the fact that everyone who reached out to the researchers met the inclusion criteria, they were included in the final sample, which resulted in a response rate of 100%. All participants completed the study without dropouts.

#### 2.4.3. Process

The first author conducted semi-structured, in-person interviews. Each participant completed two interviews, conducted three to six months apart, with each interview lasting one to three hours. The second interview aimed to clarify and further elaborate on issues that emerged during the preliminary analysis of the first interview. It also provided participants with the opportunity to expand upon or refine their accounts where they considered this appropriate. This process enhanced the credibility of the findings by ensuring that the researchers’ interpretations accurately reflected participants’ perspectives rather than assessing participants’ recall of their initial interview. Interviews were not limited by time, and only participants and researchers were present. Most of the interviews were conducted at home, at the will of the participants, and no one else was present during the process. The focus of the interviews was on participants’ insights, expectations and their experiences following their child’s suicide. Thus, they were encouraged to discuss their feelings and perceptions about the suicide and subsequent concerns.

The interview guide used for data collection has been previously published by Zavrou et al. [2].

At the first interview, participants’ demographic information along with details about the child’s suicide was collected. This information was documented on a data sheet.

### 2.5. Data Analysis

To gain a comprehensive understanding, each interview was read multiple times and was then individually analyzed by each researcher (RZ, AK, and MK). Aiming to identify initial codes in accordance with the study’s research objectives, the researchers highlighted participants’ insights on (a) their needs surviving their child’s suicide (emotions, feelings, thoughts and behaviors) described as expectations, obstacles/barriers or eliminations in fulfilling everyday goals or function, and (b) experiences in accessing formal support, also described as expectations, obstacles, barriers or eliminations in relation to mental healthcare professionals/services. Special focus was given on participants’ interpretations about their interactions with healthcare clinicians/systems.

Descriptive definitions for primary codes, sub-categories and categories were developed to establish criteria for data selection. Consequently, initial groups of constructs were developed, employing an inductive method rather than adhering to any specific theoretical framework. The levels of abstraction were identified to reflect additional relevant codes, sub-categories and categories. Analyzing each interview, codes, sub-categories and categories were revised, maintaining an active process of subsuming earlier categories and creating new ones [43]. The researchers conferred about their interpretations of the data to determine overarching themes, which were then described and summarized using the “one sheet of paper” method [44].

Regarding thematic saturation, data analysis proceeded concurrently with the sequential inclusion of interviews in the secondary analysis. Specifically, each interview was analyzed in relation to the new research questions, and before the subsequent interview was included in the analytical sample. Following analysis of the eighth interview, no substantially new codes were identified. Two additional interviews were then analyzed to confirm the stability and completeness of the emerging coding framework. Analysis of the last two interviews did not yield any new categories or conceptual insights. Therefore, the analysis concluded after ten interviews, consistent with the principle of theoretical saturation rather than the attainment of a predetermined sample size.

The research team determined that a sub-category and a category appearing in eighty percent of the transcripts would be deemed saturated [45]. This approach not only validated the themes included in the findings but also subtly suggested issues for further research that were rarely addressed in the first or second interviews.

Munhall’s nine criteria for research were used to assess the rigor of the study [40,46] (Supplementary Table S2). This comprehensive approach ensured that the findings were both credible and reliable, providing a robust framework for understanding the living experiences of bereaved mothers.

### 2.6. Researchers’ Positionality

At the time of the research, the RZ was a doctoral student in nursing with clinical experience in conducting suicide assessments in an inpatient psychiatric hospital. She had mastered the interviewing technique under the guidance and supervision of MK, an associate professor in mental health nursing. The data analysis team included RZ, MK and AK. MK is trained in advanced clinical and qualitative research and experienced in interview analysis. AK is a clinical psychologist with extended experience in parents’ bereavement support and qualitative data analysis.

### 2.7. Researchers’ Reflexivity

The researchers recognized that their professional backgrounds in mental health and suicide research inevitably influenced the analytic process. Throughout the analysis, they sought to remain close to participants’ narratives while discussing emerging interpretations within the research team to minimize individual assumptions. As this was a secondary analysis, RZ and MK, who had conducted the original interviews, provided valuable contextual familiarity with participants’ narratives while also supporting continuous reflection to distinguish new interpretations from those generated during the primary study.

### 2.8. Ethical Issues

The study’s procedures and confidentiality policies were explained to the participants both verbally and in writing via informed consent documents. Specifically, the research team provided participants with an information sheet detailing the study’s objectives and methodology, confidentiality, informed consent procedures, and the voluntary nature of participation, in accordance with Cypriot human research ethics regulations. Additionally, the researcher offered to discuss the study design over the phone with each participant. Written consent was obtained from all participants prior to their involvement in the study. Participants were clearly assured that no identifiable information would be disclosed at any stage of the investigation. All interviews were recorded with the participants’ consent. At each stage, participants agreed to continue to the subsequent phases of the study.

Because the interviews concerned possibly traumatic bereavement experiences, they were conducted by experienced qualitative researchers (RZ, MK) with expertise in interviewing on sensitive topics. Both researchers were advanced clinical practitioners in mental health nursing and continuously monitored participants for signs of emotional distress throughout the interviews. Participants were informed that they could pause or terminate the interview at any time without consequence. When participants appeared distressed, interviews were paused to allow emotional recovery before the participants and researcher jointly decided whether to continue. Following each interview, participants received information regarding available public mental health services, together with contact information for clinicians collaborating with the research team. Specifically, each participant was provided with a list of all public mental health resources and information about the team’s affiliated clinicians who could offer support if needed. No participant required emergency psychological intervention during data collection or in the follow-up period, as confirmed through a follow-up telephone call conducted by the research team. Moreover, the two primary researchers (RZ, MK) had access to ongoing clinical consultation with AK, a clinical psychologist and member of the research team, to discuss any issues arising during data collection or in the follow-up period. Interestingly, many participants described their interviewing experience as therapeutic, which helped alleviate the team’s initial concerns regarding participants’ well-being.

Given the emotionally sensitive nature of interviewing mothers bereaved by suicide, ethical considerations extended beyond participant care to include the well-being of the research team. The primary researcher (RZ) received regular supervision from MK, a senior mental health nurse and experienced academic with extensive expertise in providing trauma-informed support. This supervision provided an essential space for clinical reflection, emotional processing, and guidance throughout the data collection and analysis phases. Additionally, the research team had access to mental health nursing professionals who were available to offer psychological support if any member experienced distress. These measures were implemented to safeguard researcher mental health and maintain ethical rigor in conducting emotionally demanding qualitative interviews.

All methods used in this study adhered to the Helsinki Declaration of 1975, as amended in 2024, and followed the ethical guidelines of the applicable national and institutional committees on human experimentation. The National Bioethics Committee of the state where the study was conducted approved the research protocol. Given that all demographic information collected and displayed comes from a population of 1,000,000, it is impossible to pinpoint any individual participant.

## 3. Results

### 3.1. Participants’ Demographic Characteristics

The demographic data of the participants are shown in Table 1. A total of ten biological mothers whose son or daughter had died by suicide were identified and recruited. The participants’ age ranged from 45 to 63 years, and the length of bereavement varied between 3 and 19 years.

### 3.2. Categories and Sub-Categories

The core of participating mothers’ described psychosocial needs was reflected in their “persistent orientation towards protection”, in which the continuing struggle to keep an enduring relational role with their dead child, through the role of the guardian of the child’s memory and dignity, was central. In parallel, participating mothers reported their enduring protective role towards themselves and their families. Thus, guardians of memory and dignity were one expression of a broader protective orientation in relation to participants’ experienced psychosocial needs, since these mothers became guardians not only of their child’s memory and dignity, but also their family’s continuity and their own dignity and identity as mothers. In more detail, this protective orientation encompassed three interconnected domains: (1) self-protection and the need for acceptance by shielding themselves from stigma, social judgment, and emotional disintegration, (2) ensuring a safe and protective environment for the surviving family by safeguarding the psychological well-being and cohesion of surviving family members, and (3) protecting the posthumous dignity and memory of the deceased child. Therefore, guardianship reflected an ongoing care process and the need for bonding after the child’s death. Overall, this protective orientation shaped how they currently experienced their relationship with their child, and how they interpreted their grief, stigma and their interaction with support sources and formal healthcare services.

Specifically, rather than seeking formal support, participants overwhelmingly turned away from mental health services, citing a lack of empathy, cultural misunderstanding, and fear of being further stigmatized. The participating mothers described numerous psychosocial concerns and subsequent self-management strategies to alleviate themselves from the impact of loss on them, which were developed independently of receiving formal support. Mental health professionals were often perceived as inadequate or even harmful, reinforcing the participants’ drive towards self-reliance and autonomy in bereavement.

In particular, the participants’ narratives revolved around their need to protect themselves and their child’s memory from stigma and negative sentiments surrounding the suicide. They described their need to protect their own personal dignity and public image from being perceived as inappropriate mothers. By revealing their deep need to overcome inappropriate societal attitudes and ensure that their child’s existence was not forgotten and remembered positively, they described various actions taken toward this goal. Most importantly, they expressed a willingness to destigmatize their situation and positively transform their societal image through activism and community service.

Furthermore, the participants underlined their effort to protect surviving family members, especially the siblings of the deceased, from the psychosocial impact of the suicide, and to support them in moving forward with their lives. This concern was also perceived as a strong motivation to protect their own well-being, including their mental and physical health, and return to normality. Ultimately, the participants derived motivation from their orientation to create a positive legacy, fostering a sense of purpose in their grief while enhancing remembrance and challenging societal perceptions of suicide.

Importantly, these protective efforts were not merely individual coping strategies but constituted an enduring protective role and a rebuilt motherhood identity that organized how the participants reconstructed meaning, maintained continuing bonds with their deceased child and the surviving family members, negotiated stigma, and engaged with support and healthcare services.

The generic categories and sub-categories identified in the study, as outlined in Table 2, are detailed below.

### 3.3. Category 1: Orientation Towards Self-Protection and the Need for Acceptance: Shielding Themselves from Stigma, Social Judgment, and Emotional Disintegration

#### 3.3.1. Need to Make Sense of the Event and Obtain Meaningful Answers

The experience of losing a child to suicide left participants with profound pain and sadness, intensifying their need to make sense of the loss and find answers to numerous unresolved questions, particularly “why?” They grappled with questions such as, “Why did he/she do this?” and “What led to this?” When answers remained elusive, participants constructed their own interpretations of the suicide, often blaming themselves or attributing it to external factors, such as a relationship breakup or the child’s possible neurobiological condition: “Sometimes I feel a big ‘why’’ […] maybe it’s our fault that me and his dad broke up.”(P5). This search for meaning often trapped participants in a cycle of suffering, as the continued pursuit of explanations prolonged their emotional pain and grief: “Sometimes I think about finding his close friends to tell me, but then I wonder if it will really make me feel any better…”(P10).

Searching for explanations appeared to represent attempts to reconstruct a coherent narrative disrupted by the incomprehensibility of suicide.

#### 3.3.2. Need to Externalize Emotions Within an Empathic Context

Most participants described a strong need to express intense negative emotions such as sorrow, pain, and grief to protect their mental and physical well-being. However, externalizing these emotions and openly discussing their distress was perceived as particularly challenging. Indeed, when they were able to express their feelings, it typically occurred in moments of privacy: “When I was alone, I was crying. I had to cry to myself, too. I shouldn’t have kept it all inside because I was about to explode.”(P3). Their reluctance to share their emotions and thoughts with others stemmed from a perceived lack of empathy within their social networks and among healthcare professionals: “The psychologist was staring at me, as if I was an alien” (P1). Despite these experiences, participants consistently emphasized their need for empathetic understanding, primarily from peers rather than friends or healthcare professionals. They highlighted the importance of being accepted and understood by others who had experienced a similar loss, allowing them to share their traumatic experiences and emotions in a safe and supportive environment: “[…] mothers who have lost a child, it’s important to cry, to laugh, to listen, and to be heard. Don’t keep everything to yourself. Spend time with others who have experienced similar losses; they can understand what you’re going through. I believe this can be helpful.” (P5). Overall, participants’ lack of trust in formal healthcare systems and subsequent preferences for peer support networks was evident. Indeed, shared living experiences appeared to confer a unique form of legitimacy, enabling participants to disclose emotions without fear of misunderstanding or judgment.

#### 3.3.3. Need for Social Withdrawal and Self-Isolation

Many participants expressed their need to withdraw from their social networks: “I shut myself up at home. Whoever wants to meet me, I said, should come to my house and see me. I have nowhere to go.”(P9). Participants’ insistence on privacy reflected their experience of bereavement as a deeply personal process that they perceived as inaccessible to others. Protecting their privacy, therefore, became a way of preserving ownership over their grief and resisting external interpretations or judgments. For some, this withdrawal reflected an expression of their mourning: “[…] I don’t go outside. I don’t have the desire or the motivation I had before the incident.”(P6).

For others, it resulted from the social stigma they experienced following their child’s suicide: “ I was told: ‘Why are you grieving for her? She was a drug addict! It’s better that she’s gone’ […] So, I stopped talking to others or hanging around with them.” (P7). In these circumstances, participants sought to shield and protect themselves from negative comments, judgement, and the lack of understanding and empathic support from others, including healthcare professionals. They described how social stigma evoked feelings of sadness, disappointment, and guilt, as society often portrayed them as inadequate mothers and implied that they were responsible for their child’s death. Such societal criticism intensified participants’ grief and contributed to their social isolation: “I used to go out […] and when I heard them saying, ‘instead of helping her son, she let him hang himself,’ I stopped going out.” (P1).

Maintaining privacy functioned for many participants as a means of regulating interpersonal boundaries and protecting participants from further emotional intrusion and harm.

#### 3.3.4. Need for Self-Reliance, Autonomy and Independence Through the Bereavement Process

Participants emphasized their need for self-reliance and autonomy throughout the bereavement process: “[…] you always need to be yourself and stand on your own two feet […] you don’t want that kind of ‘coddling’” (P1). Relying on others during bereavement was often perceived as a threat to their self-respect and personal autonomy. In contrast, maintaining autonomy appeared to protect participants’ identity and dignity following a traumatic event that had challenged their roles as mothers and disrupted their assumptions about themselves and the world.

Furthermore, this need to protect their independence was reinforced by experiences of disempowering attitudes within their social network and encounters with healthcare professionals. It also reflected participants’ need to grieve in their own way, while avoiding potential harm arising from judgment, intrusiveness, or inappropriate responses from others: “I struggled with this for 6–7 months until I managed to do psychotherapy on my own; talked to myself, and got back on my feet. I didn’t take any sedative, nor did I see a doctor.” (P3). Participants’ encounters with healthcare professionals appeared to shape their broader trust in formal support systems, influencing subsequent help-seeking and reinforcing preferences for self-reliance.

Overall, lack of empathic support, coupled with the need to protect their self-respect and autonomy, led participants to rely on their own strengths as a means of protecting themselves from further emotional distress. These accounts illustrated how participants’ perceptions of healthcare professionals, together with the stigma surrounding suicide, contributed to their disengagement from healthcare services, despite their profound psychological needs.

The need for independence was also accompanied by participants’ need for privacy and the protection of their personal lives. Participants described struggling to protect their privacy and preserve their own ways of managing the impact of their child’s suicide: “I’m not even obliged to justify myself to anyone. Each house knows how it goes.”(P8). Consequently, they emphasized that the way they lived and the choices they made were inherently personal matters and that they were not accountable to anyone else, as they were the only ones who truly understood their own experiences and struggles. Nevertheless, this need for autonomy and self-determination was essential to their emotional well-being and personal recovery.

#### 3.3.5. Need to Physically and Mentally Escape

Participants also expressed their need to escape the routines, thoughts, and memories associated with their child’s suicide. Overwhelmed by these thoughts and driven by a strong need for recovery, they often withdrew from their social environment and formal mental healthcare services. Although this response functioned as a coping strategy, it also reflected their response to inadequate care provided by mental health clinicians: “[…] the psychologist was crying, devastated by my experience of my child’s suicide, along with the death of my husband. I never visited her again […].”(P1). Such inappropriate responses further contributed to participants’ disengagement from healthcare systems despite their profound psychological distress and unmet mental health and psychological needs. Moreover, these narratives suggested that formal healthcare was experienced not merely as unavailable but as emotionally inaccessible. Participants described encounters in which the absence of acknowledgement and professionalism reinforced feelings of isolation, indicating that unmet needs extended beyond service availability to the quality of relational care.

To cope, participants adopted various strategies that helped them distance themselves from the traumatic event. They described finding temporary relief and a sense of rejuvenation through activities such as traveling, walking in nature, or visiting the cemetery. These activities offered a temporary escape from painful memories, fostered a comforting connection with their emotions, and helped them manage the emotional burden of their grief: “ […] You want, let’s say, a condition where you can escape your own bad memories. To do pleasant things […] I preferred to be away from home, and away from the city, even away from the country, to stay far away” (P1).

Temporary withdrawal from suicide-related reminders appeared to facilitate emotional regulation by creating psychological distance from overwhelming grief.

### 3.4. Category 2: Orientation Towards Ensuring a Safe and Protective Environment for the Surviving Family: Safeguarding the Psychological Well-Being and Cohesion of Surviving Family Members

#### 3.4.1. Need to Protect Surviving Family Members

Most participants reported engaging in protective behaviors towards surviving family members, including spouses, surviving siblings, or grandchildren. They made deliberate efforts to support them and attend to their needs, with the aim of creating a safe environment, shielded from the negative emotions and circumstances surrounding the suicide: “We try not to show our pain […] what we go through, we go through it on our own, so our children won’t notice and feel sad […]”(P10).

As a result, participating mothers avoided discussing the suicide and refrained from externalizing their grief in the presence of surviving family members. This approach was primarily intended to protect surviving siblings by creating an environment that was, as much as possible, shielded from the emotional impact of the suicide. In doing so, they sought to spare their family members additional emotional burden and prevent further distress within the family. The following quotation clearly illustrated how participating mothers continued to prioritize others’ emotional needs over their own, preserving their maternal identity through caregiving despite experiencing profound grief: “I don’t show my (surviving) children how I feel when I‘m alone, thinking about the suicide. When I’m with my (surviving) children I am a different person. I don’t want to make them feel sad.” (P9). Relevant quotations also suggested that emotional concealment was an intentional relational strategy rather than simply emotional avoidance. Importantly, through these narratives it was evident that motherhood did not end with the child’s death. Rather, participants appeared to reconstruct their maternal role by redirecting caregiving toward surviving family members.

#### 3.4.2. Need to Protect Family Cohesion

Participants’ need to preserve family cohesion emerged as an extension of their broader desire to protect surviving family members from the devastating and disruptive impact of suicide. Their narratives reflected fears of potential family fragmentation and the loss of emotional connectedness, both within the family as a whole and among individual members. Special emphasis was placed on the surviving siblings, with participants describing deliberate efforts to ensure that they were not negatively impacted by the suicide or placed at increased risk of suicidal behavior similar to that of the deceased sibling. This protective stance underscored their determination to safeguard the well-being and mental health of the entire family while maintaining a supportive and cohesive family environment: “I wanted the surviving family members close to me. I felt a very strong need to be near them […] to do things for them and support them “ (P3). Keeping surviving family members close appeared to represent an effort to restore a sense of control and safety following the unpredictability and trauma of the suicide.

Overall, participants’ accounts reflected a need to support and strengthen their family in the aftermath of their child’s suicide. Central to this was their desire to protect family members, shield them from further emotional suffering, and preserve family unity. Nevertheless, protecting the family was not only a moral obligation for the participating mothers, but also provided them with renewed purpose and a perception of responsibility, which became psychologically restorative. Assuming responsibility for the family’s well-being appeared to restore participants’ sense of agency following the devastating loss of control associated with suicide bereavement.

This is also evident through narratives which showed that protecting surviving family members and maintaining family cohesion simultaneously served as a strong motivation for participants to protect their own mental and physical well-being, and gradually restore a sense of normality. As one participant explained: “[…] I kept telling myself, ‘You are just fine’ […] I had to support the whole family […] This made me very strong […].” (P3).

At the same time, protecting surviving family members functioned as a mechanism of self-preservation, enabling participants to regain emotional stability, maintain hope, and continue functioning despite overwhelming grief.

### 3.5. Category 3: Orientation Towards Protecting the Posthumous Dignity and Memory of the Deceased Child

#### Need to Release Their Child from the Stigma and Themselves from the Guilt Related to the Suicide

Participating mothers often sought to protect their child’s memory from the stigma associated with suicide, particularly the perception that suicide reflected a flawed or morally compromised character. They expressed a deep need to ensure that their child would not be forgotten and, above all, would be remembered positively. They achieved this by keeping photographs, preserving personal belongings, and often speaking about their child in the present tense: “When I see his things, they bring me closer to him. I have many of his clothes in the closet, and his younger brother wears them too, so he isn’t forgotten.” (P6). These practices reflected more than acts of remembrance; they represented efforts to preserve an ongoing bond with their child and to reclaim their child’s identity from the stigma surrounding suicide. Rather than allowing the suicide to define their child, participating mothers actively maintained memories that emphasized their child’s continuing place within the family and their life beyond the circumstances of death.

Participants also described finding strength and motivation to continue with their lives through the hope of helping others in need in their child’s memory, for example by engaging in charitable activities. These actions enabled them to create a lasting and positive legacy associated with their child’s name. In doing so, they sought not only to preserve their child’s memory but also to challenge the negative social meanings attached to suicide and by reclaiming a more positive narrative of their child’s life: “[…] this is what gives me the strength to live and do things […] I want to live to do things in memory of my child.”(P2).

Creating a positive legacy also appeared to help participants cope with feelings of guilt associated with their child’s death by transforming painful emotions into meaningful actions. By preserving memories and engaging in meaningful acts of remembrance, they found a renewed sense of purpose while actively resisting the stigma surrounding suicide. These efforts not only honored their child’s memory but also represented attempts to restore both their child’s social identity and their own identity as mothers, resisting societal narratives that reduced their child solely to the suicide. Consequently, preserving their child’s memory became both an act of remembrance and a means of meaning-making, enabling participants to cope with their grief while symbolically freeing their child’s memory from the stigma associated with suicide.

Overall, the categories described suggest that the participants’ responses did not represent isolated coping strategies but rather interconnected manifestations of a broader psychosocial process characterized by an enduring orientation towards restoring safety, preserving identity, and protecting the deceased child, themselves and their families following the suicide. This overarching process appeared to be strongly influenced by culturally embedded expectations surrounding motherhood, family responsibility, and suicide-related stigma.

## 4. Discussion

This study identified the central need of the participating suicide-bereaved mothers: their enduring protective orientation towards their deceased child, themselves and their families. This protective alignment shaped not only how they interpreted their grief, experiences of stigma and how they engaged with informal support and formal healthcare services, but also how the participating mothers experienced their continuing relationship with their child and the surviving family members. Importantly, these findings suggest for the first time that this protective orientation functioned not merely as a coping strategy but as an organized principle through which the bereaved participating mothers reconstructed meaning, maintained continuing bonds with their deceased child and the surviving family members, and negotiated a stigmatizing social environment, including healthcare settings. This perspective offers a novel contribution by integrating these processes within a single interpretative framework, which provides a model of post-suicide maternal identity reconstruction under the experiences of loss and stigma; an identity which reflects a relational and moral protective role. Indeed, through the guardianship of the child’s memory and dignity, these mothers maintained an enduring relational role with their deceased child, constructing a new dimension of their motherhood identity and at the same time elaborating a transformed relationship with their deceased child.

Furthermore, this study contributed new insights to the literature on women’s grief in Southern Europe and the Eastern Mediterranean not only by providing data on the expressed psychosocial needs of suicide-bereaved mothers, but also on the difficulties in receiving formal support faced by Greek-speaking mothers in the RC. Specifically, these findings showed that participating mothers did not avoid support because they sought isolation. Rather, they withdrew from support that failed to provide psychological safety. Consequently, help-seeking depended on whether support was perceived as emotionally safe, empathic, and free from judgment and criticism.

To date, very few studies have addressed women’s suicide bereavement in this particular context, since prior research has largely focused on Anglo-Saxon and East Asian settings, often with mixed-gender samples [25]; such study designs risk obscuring gender-specific dynamics of grief and coping [15,24,33]. Our findings extended existing knowledge [2,17,32,47], by showing how culturally and gendered-oriented expectations shape mothers’ priorities, concerns and bereavement-related self-management strategies, including (a) the reconstruction of their motherhood identity, (b) the revelation of new dimensions of their need to keep alive the relationship with their deceased child, (c) the challenges they face in accessing formal mental healthcare, and (d) their orientation to fulfill their psychosocial needs. Overall, this study is among the few that revealed what suicide-bereaved mothers were striving to accomplish after the suicide.

Based on the present results, participants’ expressed psychosocial needs cantered on a “persistent orientation towards protection” encompassing three interconnected domains: (1) self-protection and the need for acceptance by shielding themselves from stigma, social judgment, and emotional disintegration, (2) ensuring a safe and protective environment for the surviving family by safeguarding the psychological well-being and cohesion of surviving family members, and (3) protecting the posthumous dignity and memory of the deceased child. These findings are consistent with studies supporting the stigmatization of suicide bereaved parents and those who died by suicide [6,48]. While our findings are consistent with previous qualitative studies reporting stigma, self-blame, and barriers to help-seeking among suicide-bereaved parents, the present study extends existing knowledge by demonstrating how these experiences are shaped by the sociocultural context of the RC and by gender-specific expectations surrounding motherhood.

Indeed, the participating mothers in the present study consistently described a strong desire to counteract societal judgments and ensure that their child was remembered positively, sometimes by engaging in charitable or community activities that served as legacy-building acts. This was partially in line with previous studies showing the importance of countering negative perceptions and keeping the child’s memory alive as part of the grieving process [25,49]. Similarly, Shields et al. [47] highlighted that bereaved mothers strive to protect the memory of their dead child, “disinfected” by the stigma of suicide.

Yet, these protective efforts may reflect culturally prescribed maternal roles, positioning mothers as the primary guardians of family cohesion and reputational honor, even while navigating profound personal grief [50]. Overall, participating mothers’ protective efforts towards surviving family members, mainly surviving siblings, highlighted family cohesion as a core need, but also positioned them as “invisible grievers”. This finding confirmed prior literature on silent grieving in suicide bereaved mothers [32], and further extended these data by revealing that mothers internalize their own distress as a strategy to shield others, especially surviving siblings, from additional emotional harm. Thus, an additional dimension of the phenomenon was highlighted, and the bereavement experience was not only described as a deeply personal psychological struggle but also as a moral and social responsibility, leading to a reconstruction of the motherhood identity and the development of new dimensions within it, mainly serving as a coping strategy and a process probably shaped by gendered cultural norms [50]. Participating mothers did not experience their loss solely as an inner emotional process; they simultaneously felt compelled to uphold family honor, shield their families from social judgment, and fulfill culturally prescribed maternal duties of care and sacrifice. Their mourning, therefore, was both an intimate process of coping with trauma and a public duty to demonstrate resilience, preserve cohesion, and defend the dignity of the deceased child against stigma. This interpretation aligns with prior research highlighting the gendered burden of grief, whereby mothers, more than fathers, are expected to sustain family unity, regulate emotions, and safeguard reputational standing in the face of loss [51,52,53]. Most importantly, the present study confirms such data for the first time in suicide-bereaved mothers in the Southern European and Eastern Mediterranean region.

Overall, these findings emphasized the influence of cultural context on maternal bereavement; the RC is characterized by close-knit communities, strong family ties, and culturally embedded norms regarding honor and reputation [30]. Within this setting, participating mothers’ selective social engagement and emphasis on autonomy can be interpreted as protective strategies against gossip, judgment, and social intrusion. While these behaviors may appear as social withdrawal, they more accurately reflect culturally specific efforts to preserve dignity and safeguard both the family’s cohesion and the deceased child’s memory, dynamics further intensified by gender social norms [54].

An additional key finding was participants’ reliance on peer support and informal networks rather than on formal mental health services during the bereavement period. Participating mothers deliberately avoided formal care, citing prior negative experiences with clinicians, perceived lack of empathy, cultural insensitivity, and fear of further stigmatization. This disengagement reflected a dynamic interplay of systemic inadequacies of healthcare services with gendered dynamics: participating mothers internalized social expectations to remain resilient and emotionally contained, while healthcare professionals often viewed them through dehumanizing stereotypes that minimized their vulnerability. As a result, participants’ intense psychosocial experiences and related expectations were left unmet, reinforcing cycles of isolation and silence. These findings echo prior data showing that bereaved mothers in patriarchal societies internalize distress, experience stigma, and believe that they have to be strong [51]. Additionally, these findings confirm earlier studies showing that women’s help-seeking behaviors are strongly influenced by stigma, social expectations of emotional strength, and negative prior experiences with healthcare systems [55].

### 4.1. Implications for Practice

These findings have important implications, as they suggest that relevant interventions must recognize that suicide-bereaved mothers often avoid formal services due to systemic, cultural and gender-related barriers. Subsequently, effective postvention strategies should prioritize peer-led, mother-specific support programs that validate grief while addressing the social and moral dimensions of suicide bereavement. Peer support groups, in particular, could reduce feelings of isolation by connecting mothers with others sharing similar loss. Sharing narratives in a compassionate, nonjudgmental setting fosters mutual understanding, reduces stigma, and provides a renewed sense of hope and control [31,56,57].

Furthermore, although engagement in activism and community service has been previously identified as a core need of suicide-bereaved family members [25], the present study has emphatically addressed its importance for suicide-bereaved mothers; the participants clearly linked such actions to their need to counteract the negative social image of their child, underscoring how maternal bereavement carries reputational stakes. While a previous meta-synthesis highlighted the importance of maintaining a non-traumatizing memory of the deceased child and participation in supportive activities [25], the present study demonstrated an additional maternal imperative, as the participants’ intense need was to ensure that their child’s memory was preserved positively. This new deeper understanding has critical implications for women’s mental health across the lifespan, particularly in conservative cultural settings where stigma strongly shapes help-seeking behavior.

Importantly, not all suicide-bereaved mothers are willing to engage in public remembrance or activism; some may prefer private, symbolic practices or even silence. Both strategies represent culturally and personally valid grief responses [58,59]. Thus, postvention programs need to be flexible, offering personalized grief pathways, ranging from private journaling to small-group peer support, or public-facing advocacy, allowing suicide-bereaved mothers to choose the form of remembrance that best aligns with their expectations and priorities [58,59].

Moreover, the participants herein expressed a strong desire to transform public perceptions of suicide and advocate for their children’s dignity. Their involvement in public awareness campaigns may thus serve a dual purpose: contributing to broader de-stigmatization efforts while providing mothers with channels for post-traumatic growth and identity reconstruction [60,61]. Survivor advocacy groups could play a key role by facilitating campaigns where mothers share their stories, framing narratives around self-compassion over blame.

While encouraging mothers’ engagement in activism is important, professional support towards them remains essential. Suicide-bereaved parents often struggle with PTSD, depression, and anxiety in addition to grief [62]. Early access to counselling, specifically tailored to suicide bereavement, may reduce guilt, self-blame, and other cognitive distortions [63]. Nevertheless, the participants herein constantly mentioned the lack of professional training on suicide bereavement. Previous studies also underscored the need for clinicians to be equipped to provide this type of care [62]. Based on the present findings, training should go beyond standard grief models; professionals need to be prepared to empathically recognize that for mothers, suicide bereavement represents not only a personal loss but also a social and reputational crisis, particularly in stigmatizing societies [58,64]. Professionals should also recognize that many mothers continue to experience themselves as protectors of their deceased child and family, making validation of this enduring maternal role a fundamental component of therapeutic engagement. Therefore, relevant training modules should include: (a) case-based scenarios highlighting mothers’ struggles with stigma, reputational concerns, and memory preservation, (b) role-playing experiences to sensitize clinicians to survivors’ fears of judgment, gossip, and community scrutiny, (c) Stigma-informed, culturally competent content, with attention to kinship ties, family relationships and collective identity in contexts similar to the RC. Developing continuing education curricula around stigma-informed care for suicide bereavement would strengthen professional capacity, foster therapeutic trust, and facilitate greater engagement with services.

Beyond professional training, the present findings suggest that suicide postvention services should be designed around the principle of psychological safety. The participating mothers did not avoid professional support because they preferred isolation; rather, they disengaged from interactions that were perceived as judgmental, stigmatizing, or emotionally unsafe. Consequently, the effectiveness of postvention services may depend not only on their availability but also on their ability to provide environments in which mothers feel accepted, respected, and able to disclose their experiences without fear of blame or social scrutiny. Healthcare organizations should, therefore, consider incorporating the promotion and evaluation of psychological safety as a core component of postvention care, alongside traditional indicators of service accessibility and quality.

Furthermore, the present findings indicate that interventions should acknowledge the enduring protective orientation that characterized the participating mothers’ experiences. Rather than conceptualizing mothers solely as recipients of bereavement support, clinicians should recognize that many continue to perceive themselves as protectors of their deceased child’s dignity, memory, and moral identity, while simultaneously safeguarding the wellbeing of surviving family members. This perspective has important implications for clinical assessment and intervention, suggesting that professionals should routinely explore concerns related to stigma, reputational worries, memory preservation, and continuing maternal responsibilities. Therapeutic approaches that facilitate narrative reconstruction, legacy-building, continuing bonds, and meaning-making may, therefore, be particularly beneficial, as they validate mothers’ ongoing relational role with their deceased child instead of focusing exclusively on grief symptom reduction.

At the service level, these findings support the development of postvention models that extend beyond conventional grief counselling by integrating stigma-informed, gender-sensitive, and family-oriented care. Because the participating mothers described their protective role as encompassing both the deceased child and the surviving family members, interventions should, where appropriate, include opportunities for family communication, shared meaning-making, and collaborative remembrance. Such approaches may strengthen family resilience while reducing the social isolation frequently experienced by suicide-bereaved mothers. More broadly, incorporating these principles into national suicide prevention and postvention strategies may contribute to more responsive services that address not only the psychological consequences of suicide bereavement but also the relational, moral, and social dimensions of maternal grief identified in the present study.

Despite these requirements, in the RC, formal postvention systems remain underdeveloped. General mental health services are available, but there are no specialized suicide-bereavement programs, no gender-sensitive interventions, and no structured peer support groups. Informal bereavement support, where it exists, typically focuses on losses due to chronic illness rather than suicide. Limited community awareness and public discourse reinforce stigma, further discouraging help-seeking. As a result, bereaved mothers often grieve in isolation, with minimal support from either state institutions or community organizations. This service gap is particularly alarming, given that in both the RC and Greece, bereaved parents due to chronic illness (e.g., cancer) do have access to specialized groups, while parents bereaved by suicide do not. This disparity reflects the intensity of stigma around suicide and illustrates how mothers. Indeed, most participants herein reported that such absence of service left them exposed to insensitive behaviors from family and friends, resulting in social isolation and rejection, findings consistent with previous data [6,31,46,48,65].

Finally, there is a strong case for establishing a registry of suicide-bereaved families in the RC. Such a tool would enable targeted interventions, ensure outreach to those at risk of isolation, and facilitate the implementation of tailored, culturally and gender relevant services.

### 4.2. Limitations and Future Research

Despite its strengths, the present study encompasses a number of limitations. While focusing exclusively on suicide-bereaved mothers allowed for an in-depth understanding of gender-specific expectations and challenges in help-seeking, this approach inevitably limited insights to fathers or mixed-gender samples. It is recommended, therefore, that future research should examine gender differences in grief, self-management strategies, and help-seeking, while also considering cultural variations. Broader sampling strategies, including online surveys or mixed-methods approaches, could capture a wide range of bereavement experiences and may provide a more comprehensive understanding of how gender and sociocultural context shape grief.

Furthermore, although participants were between three and nineteen years bereaved, the present study did not compare experiences across stages of bereavement. Although this is clinically relevant, the present study was not designed to compare experiences across stages of bereavement. Nevertheless, narratives suggested that certain needs persisted regardless of time since loss, particularly the need for validation and non-judgmental professional support. Future longitudinal research should investigate how mothers’ needs evolve over time.

Another limitation concerns methodological reporting. While the current study adhered to the COREQ checklist to enhance transparency and completeness [42], we acknowledge the ongoing scholarly debate regarding COREQ’s credibility and applicability. As Braun and Clarke [66] have argued, COREQ should not be treated as a universally applicable standard for qualitative research, particularly when applied without attention to methodological congruence. In light of these critiques, we clarify that our use of COREQ was intended to support transparency rather than to substitute rigor. The design and implementation of this study were grounded in inductive content analysis and guided by principles of methodological coherence, reflexivity, and transparency [45]. Additionally, Munhall’s nine criteria for qualitative search [46] were used to assess rigor. Thus, the COREQ checklist was used as a reporting scaffold rather than a methodological template. Future studies may benefit from more critically tailoring their reporting so they align more closely with the epistemological and methodological paradigms underpinning their design.

## 5. Conclusions

This study supported that participating suicide-bereaved mothers experienced profound, gender-specific needs centered on protecting themselves, their child’s memory and dignity, and family cohesion. Despite intense psychological distress, stigma and societal expectations limited their engagement with formal mental health services. Peer support, culturally competent interventions, and legacy-focused programs are expected to provide promising avenues to meet these needs, emphasizing the importance of gender-informed and culturally sensitive postvention care. Recognizing suicide-bereaved mothers’ enduring protective role and validating both their private and public coping strategies may serve as a foundation for more effective interventions that support mothers’ ability to navigate grief while safeguarding their child’s legacy and family integrity.

Importantly, the originality of this study lies in demonstrating that these protective efforts are not merely individual coping strategies but constitute an enduring protective role and a rebuilt motherhood identity that organizes how suicide-bereaved mothers reconstruct meaning, maintain continuing bonds with their deceased child and the surviving family members, negotiate stigma, and engage with support and healthcare services. Overall, this study extends previous research by demonstrating that the principal challenge faced by suicide-bereaved mothers is not only grief itself but the experience of that grief within healthcare systems, which often fail to recognize their unique emotional and relational needs. Thus, the findings provide clinically relevant evidence for developing gender-sensitive, stigma-informed postvention services capable of supporting mothers across the lifespan following the suicide of their child.

## Figures and Tables

**Table 1:** Demographic variables of the participants and their child who died by suicide.

A. Demographic Characteristics of the Child Who Died by Suicide.						B. Demographic Characteristics of the Participating Mothers.					
Participant	Sex	Age (Years)	Method of Suicide	Place of Suicide	Number of Siblings	Sex	Age (Years)	Marital Status	Years Away from Suicide	Religion	Occupation
1.	Male	34	Hanging	Home	None	Female	63	Widow	15 years	Chr. Orthodox	Retired
2.	Male	22	Hanging	Home	None	Female	45	Divorced	4 years	Chr. Orthodox	Private sector employee
3.	Female	16	Drugs ingestion	Home	One	Female	62	Married	19 years	Agnostic	State sector employee
4.	Male	23	Leap into space	Near	One	Female	59	Widow	5 years	Chr. Orthodox	Unemployed
5.	Male	23	Self-shot	Home	Two	Female	54	Divorced	3 years	Chr. Orthodox	Unemployed
6.	Male	30	Drugs ingestion	Home	One	Female	59	Divorced	12 years	Chr. Orthodox	Private sector employee
7.	Male	28	Leap into space	Near home	Two	Female	60	Widow	5 years	Chr. Orthodox	Retired
8.	Male	40	Poisoning	Home	Two	Female	63	Widow	16 years	Chr. Orthodox	Retired
9.	Male	28	Self-shot	Near home	One	Female	56	Married	7 years	Chr. Orthodox	Private sector employee
10.	Male	23	Hanging	Home	Two	Female	55	Married	8 years	Chr. Orthodox	Unemployed

**Table 2:** Identified core theme, categories/sub-categories and codes.

Main Category (Core Theme)	Categories (Generic)	Sub-Categories	Codes
**Persistent orientation towards protection**	**Orientation towards self-protection and the need for acceptance: shielding themselves from stigma, social judgment, and emotional disintegration**	**Need to make sense of the event and obtain meaningful answers.**	Need for answers Need for identifying the cause of suicide Need for meaning
		**Need to externalize emotions within an empathic context.**	Need to express emotions Need for empathic understanding Difficulty in sharing emotions Need for acceptance from peers
		**Need for social withdrawal** **and self-isolation.**	Voluntary isolation Social withdrawal due to stigmatization Protection strategies from negative comments/criticism
		**Need for self-reliance, autonomy and independence through the bereavement process.**	Avoiding reliance on others/healthcare professionals Self-help strategies Protection of personal autonomy/independence Protection of privacy
		**Need to physically and mentally escape.**	Need for physical and mental way out Relief through distraction Response to inadequate mental healthcare
	**Orientation towards ensuring a safe and protective environment for the surviving family: safeguarding the psychological well-being and cohesion of surviving family members**	**Need to protect surviving family members.**	Protective behaviors towards surviving family members: spouse, siblings, grandchildren Avoiding externalization of emotions to prevent further distress within the family Protecting surviving siblings from suicidality
		**Need to protect family cohesion.**	Efforts to maintain family unity Emotional shielding behaviors
	**Orientation towards protecting the posthumous dignity and memory of the deceased child.**	**Need to release their child from the stigma and themselves from the guilt related to the suicide.**	Actions to protect the child’s memory from suicide-related stigma Actions to maintain the child’s memory Charitable activities Coping mechanism for relief

**Note:** Bold text indicates the generic categories, while regular text indicates the sub-categories.

## Data Availability

The participants in this study did not consent to have their complete transcripts made publicly available after data analysis, as containing personal information for which they could be identified. Despite the anonymization of the interview transcripts, there is always danger of breach of confidence. Therefore, the data underlying the results presented in the study are only available internally, and interested parties are advised to contact the corresponding author. Finally, the authors attest that the manuscripts have the information needed to support the findings of the study.

## Supplementary Materials

The following supporting information can be downloaded at: https://www.mdpi.com/article/10.3390/nursrep16080262/s1, Table S1: The consolidated criteria for reporting qualitative research (COREQ) checklist; Table S2: Presentation of the criteria applied to ensure the rigour and trustworthiness of the study.

## Public Involvement Statement

No public involvement in any aspect of this research.

## Guidelines and Standards Statement

This manuscript was drafted in accordance with the Consolidated Criteria for Reporting Qualitative Research (COREQ) checklist (Tong, Sainsbury, and Craig, 2007 [42]) for qualitative interview-based research.
