# Supplementary material for: Guardians of Memory, Dignity, and Family Cohesion: The Enduring Protective Role Underpinning Suicide-Bereaved Mothers’ Psychosocial Needs and Engagement with Mental Health Care
Source: Nurs Rep. 2026 Jul 29;16(8):262. doi: 10.3390/nursrep16080262 (PMC13516671; doi:10.3390/nursrep16080262)
Supplement: Supplementary file 1 [file nursrep-16-00262-s001.zip › nursrep-4390935-supplementary.pdf]

SUPPLEMENTARY TABLE S1: *The consolidated criteria for reporting qualitative research (coreq) checklist*

COREQ (COnsolidated criteria for REporting Qualitative research) Checklist

Topic	Item No.	Guide Questions/Description	Reported on Page No.
**Domain 1: Research team and reflexivity**			
Personal characteristics			
Interviewer/facilitator	1	Which author/s conducted the interview or focus group?	8
Credentials	2	What were the researcher's credentials? E.g. PhD, MD	8
Occupation	3	What was their occupation at the time of the study?	8
Gender	4	Was the researcher male or female?	8
Experience and training	5	What experience or training did the researcher have?	8
Relationship with participants			
Relationship established	6	Was a relationship established prior to study commencement?	9
Participant knowledge of the interviewer	7	What did the participants know about the researcher? e.g. personal goals, reasons for doing the research	8, 9
Interviewer characteristics	8	What characteristics were reported about the inter viewer/facilitator? e.g. Bias, assumptions, reasons and interests in the research topic	9
**Domain 2: Study design**			
Theoretical framework			
Methodological orientation and Theory	9	What methodological orientation was stated to underpin the study? e.g. grounded theory, discourse analysis, ethnography, phenomenology, content analysis	6
Participant selection			
Sampling	10	How were participants selected? e.g. purposive, convenience, consecutive, snowball	7
Method of approach	11	How were participants approached? e.g. face-to-face, telephone, mail, email	7
Sample size	12	How many participants were in the study?	7
Non-participation	13	How many people refused to participate or dropped out? Reasons?	8
Setting			
Setting of data collection	14	Where was the data collected? e.g. home, clinic, workplace	9
Presence of non-participants	15	Was anyone else present besides the participants and researchers?	9
Description of sample	16	What are the important characteristics of the sample? e.g. demographic data, date	9
Data collection			
Interview guide	17	Were questions, prompts, guides provided by the authors? Was it pilot tested?	9
Repeat interviews	18	Were repeat inter views carried out? If yes, how many?	9
Audio/visual recording	19	Did the research use audio or visual recording to collect the data?	10
Field notes	20	Were field notes made during and/or after the inter view or focus group?	no
Duration	21	What was the duration of the inter views or focus group?	9
Data saturation	22	Was data saturation discussed?	8, 12
Transcripts returned	23	Were transcripts returned to participants for comment and/or	

Topic	Item No.	Guide Questions/Description	Reported on Page No.
		correction?	9
**Domain 3: analysis and findings**			
Data analysis			
Number of data coders	24	How many data coders coded the data?	11-18, Table 2
Description of the coding tree	25	Did authors provide a description of the coding tree?	Table 2
Derivation of themes	26	Were themes identified in advance or derived from the data?	data
Software	27	What software, if applicable, was used to manage the data?	no
Participant checking	28	Did participants provide feedback on the findings?	no
Reporting			
Quotations presented	29	Were participant quotations presented to illustrate the themes/findings? Was each quotation identified? e.g. participant number	12-18
Data and findings consistent	30	Was there consistency between the data presented and the findings?	12-18, Table 2
Clarity of major themes	31	Were major themes clearly presented in the findings?	11, Table 2
Clarity of minor themes	32	Is there a description of diverse cases or discussion of minor themes?	12-18

Developed from: Tong A, Sainsbury P, Craig J. Consolidated criteria for reporting qualitative research (COREQ): a 32-item checklist for interviews and focus groups. *International Journal for Quality in Health Care*. 2007. Volume 19, Number 6: pp. 349 – 357

SUPPLEMENTARY TABLE S2. *Presentation of the criteria applied to ensure the rigour and trustworthiness of the study.*

Criterion	Description	How the criterion was supported in the study
Reasonableness	The results constitute a reasonable interpretation of the phenomenon	The logic of the findings was confirmed in the phase of repeated interviews and presentation of the findings to the participants through the participants' expressions: "Yes, it seems logical to me", "Yes, it makes sense to me". The integrity of data analysis process was assured by continuous meetings of the three researchers in order to determine the degree of consensus among them regarding the findings. In case of a disagreement

		extensive discussion was followed until a consensus among the researchers was achieved, and all three researchers came into a consensus regarding study findings.
Resonance	The analysis of the phenomenon reverberates with participants	In the phase of repeat interviews and the presentation of the findings to the participants, they confirmed the analysis using expressions such as: “Yes it sounds right to me”
Representativeness	In depth interpretation of the phenomenon	Data collection was finalized when it was found that the addition of further data contributed significantly to the disclosure of new data.<br>Moreover, aiming to assure data saturation during data collection process, (a) the participants were encouragement to refer to examples of their experience to enable an in depth understanding of their meaning, (b) each interview was terminated when the participants thought they had nothing to add, in answer to a relevant question, i.e. “Would you like to add anything more?”, (c) a second interview was conducted with each participant; thus, participants had the chance to reflect on their first interview and to revise, or even enrich its content, (d)

		all the participants were at least two year away from the suicide, thus able to reflect on the phenomenon.
Relevance	The relevance of the study to the objectives of the qualitative paradigm	The conclusion of the study refers to the way parents who lost a child by suicide experience this phenomenon, based on their narratives and self-perceptions.
Recognizability	Readers recognize aspects of their experience in the interpretation	The recognizability of the findings was confirmed through the presentation of these findings to mourners from another cause of death and to non-mourners, citing expressions such as: "What you are describing to me, I have seen it happen."
Raised Consciousness	Enlarging one’s perception of reality	Becoming familiar with the experience of the participants was felt as a dynamic process by the researchers; The participants narratives shed some light to the needs of this population group, but also helped the researchers to enhance their empathy towards this group since they encounter with some dimensions of the phenomenon that they had never realized before.
Readability	Feasibility and accuracy of the text	The researchers used a naturalistic was of writing the text, based on the participants narratives.
Responsiveness	The way in which the study’s participants responded	The participants confirmed both the accuracy of the descriptions and the interpretation of the phenomenon as carried out by the researcher, as well

		as the representativeness of the data compared to the way they experienced the described phenomenon.
Resonsibility	The degree to which ethical issues have been encountered.	The study protocol was approved by the national bioethics committee of the State & the the Office of Personal Data Protection.<br>Participants' consent was confirmed throughout the study.<br>Anonymity and confidentiality was ensured by the use of pseudonyms by the participants. The participants were encouraged to speak to the extent that they felt comfortable doing so.<br>The final part of the text was presented to the participants in order to confirm their consent to use quota from their interviews in the text.
